# Onset of multiple sclerosis in a patient on dupilumab therapy for atopic dermatitis: A case report

**DOI:** 10.1111/dth.15740

**Published:** 2022-08-08

**Authors:** Federica Gelato, Luca Mastorino, Paola Cavalla, Pietro Quaglino, Giovanni Cavaliere, Marco Vercellino, Chiara Bosa, Matilde Inglese, Raffaele De Palma, Michela Ortoncelli, Simone Ribero

**Affiliations:** ^1^ Dermatology Clinic, Department of Medical Sciences University of Turin Turin Italy; ^2^ Department of Neurosciences and Mental Health City of Health and Science University Hospital of Turin Turin Italy; ^3^ Department of Neurosciences University of Turin Turin Italy; ^4^ Department of Neurosciences, Rehabilitation, Ophthalmology, Genetics, Maternal and Child Health and Center of Excellence for Biomedical Research University of Genoa and IRCCS Ospedale Policlinico San Martino Genoa Italy; ^5^ Division of Clinical Immunology, Department of Internal Medicine (DIMI) University of Genoa and IRCCS Ospedale Policlinico San Martino Genoa Italy

Dear Editor,

1

A 21‐year‐old male patient affected by severe atopic dermatitis (AD) since childhood started dupilumab in January 2019 after treatment failure with cyclosporin. Serum examination at the baseline revealed high levels of IgE (6.027 IU/ml) and lactate dehydrogenase (LDH 482 IU/L). The Eczema Area and Severity Index (EASI) was 24, while the Dermatology Life Quality Index (DLQI) was 20. He reported severe pruritus (numerical rating scale [NRS] 10/10) and sleep disorders. After 16 weeks of treatment, the patient's outcomes were significantly improved with NRS of 0, EASI and DLQI of 0 and 2, respectively, IgE of 831 IU/ml and LDH of 168 IU/L.

In July 2020, he developed isolated paraesthesias in his left hand, followed by gait ataxia, and in September 2020 Lhermitte's sign and right lower limb paresis. Therefore, he was admitted at the Neurology clinic for further evaluation. The neurological examination revealed left upper limb downward drift and pronation, right leg downward drift and ankle clonus, paresthesias in the distal phalanges of the fingers of the right hand, hypopallesthesia in the left hand and right lower limb and positive Lhermitte's sign.

Magnetic resonance imaging (MRI) of the spine showed non‐enhancing spinal cord lesions at C2, C4, C5‐C6, T1, T5‐T6, and T10‐T11 (Figure 1A). Brain MRI revealed multiple non‐enhancing white matter lesions in the periventricular white matter, in the genu of the corpus callosum, in the left internal capsule, in the left temporal lobe, in the cerebellar hemispheres, and in the ponto‐mesencephalic junction (Figure 1B). Cerebrospinal fluid (CSF) analysis demonstrated mild lymphocytic pleocytosis, increased IgG index and CSF‐restricted oligoclonal bands.

**FIGURE 1:**
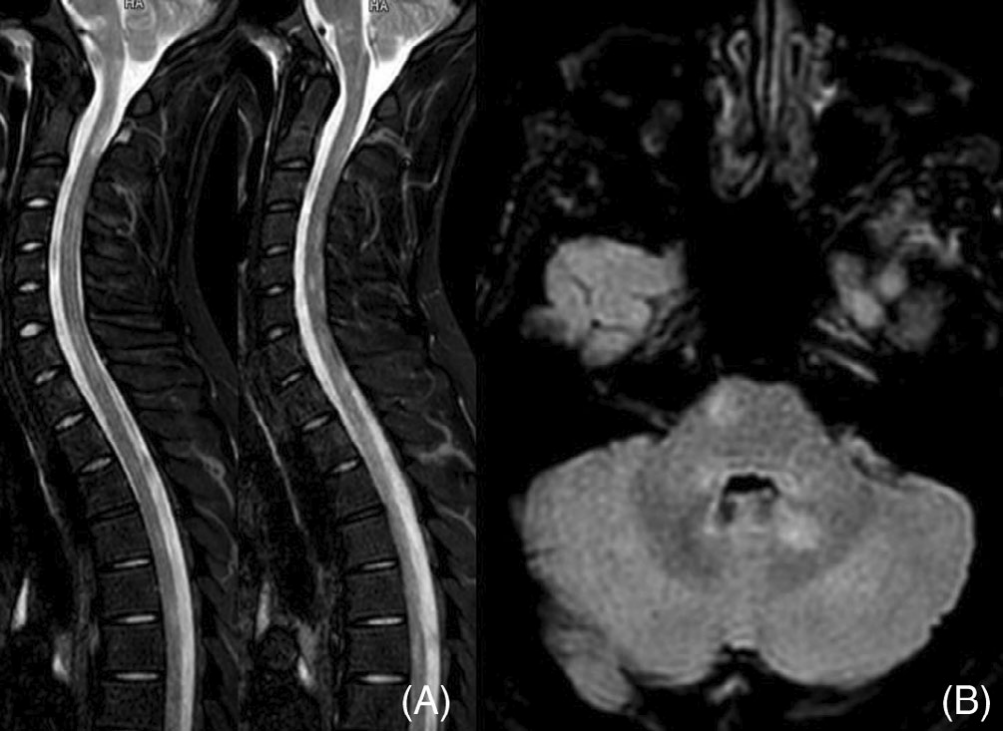
(A) Spinal magnetic resonance imaging (MRI) shows non‐enhancing spinal cord lesions at C2, C4, C5‐C6, T1, T5‐T6, and T10‐T1. (B) Brain MRI, imaging infratentorial region, shows multiple non‐enhancing lesions in the pons and in cerebellar hemispheres

Serum Aquaporin‐4 (AQP4) and myelin oligodendrocyte glycoprotein (MOG) antibodies were absent. Antibodies to Borrelia burgdorferi, Treponema pallidum, and HIV were negative. JCV Stratify Assay® tested negative.

Based on clinical history, MRI, and CSF examination, a diagnosis of relapsing–remitting multiple sclerosis (MS) was made.

Due to the aggressive presentation of MS, in March 2021 the patient was started on natalizumab (an anti‐VLA4 monoclonal antibody). As there are no data on the safety of the association with natalizumab, dupilumab was discontinued.

Since then, the patient has been clinically stable, with improvement of previous neurological symptoms and no further MS relapses. Follow‐up MRIs after initiation of natalizumab showed no further evidence of disease activity. Natalizumab therapy was well tolerated. He had moderate relapses of AD managed with topical corticosteroids alone.

AD is characterized by a Th2 cellular‐mediated immune response by cytokines IL‐13 and Il‐4.^1^ It has also been reported that Th17‐related cytokines (IL‐17 and IL‐22) and Th1 cytokines such as interferon‐γ (INFγ) play a minor role in the pathogenesis of AD.^2^

Recent studies have shown that IL‐4 has a negative regulatory action on the Th1 and Th17 pathway by silencing of IL‐23 in antigen‐presenting cells.^3^

In MS, the main mediators of inflammation are Th1 and Th17 related cytokines, such as tumor necrosis factor‐*α* (TNF*α*), INF‐γ, IL‐2, and IL‐17.^4^

We hypothesize that dupilumab, which leads to Th2 suppression by blocking the shared receptor subunit for IL‐4 and IL‐13,^5^ could lead to a shift in the immune response promoting Th1/Th17 polarization. Therefore, the use of dupilumab in genetically predisposed individuals may be a trigger event towards the development or worsening of Th1/Th17 related diseases, such as psoriasis^6^ and MS.

This case is the second reporting the onset of MS after initiation of dupilumab: Laageide et al. described the onset of similar symptoms just 2 months after initiation of dupilumab.^7^

Further studies are needed to better understand if dupilumab can increase the risk of MS onset or flareups. Meanwhile we recommend caution in prescribing dupilumab in patients with MS or a family history of MS.

## AUTHOR CONTRIBUTIONS

Federica Gelato and Luca Mastorino have given substantial contributions to the conception and the design of the manuscript; Michela Ortoncelli, Giovanni Cavaliere, and Paola Cavalla to acquisition, analysis, and interpretation of the data; Marco Vercellino, Chiara Bosa, Matilde Inglese, Raffaele De Palma have been involved in drafting the manuscript; Simone Ribero and Pietro Quaglino revised the manuscript critically. All authors have read and agreed to the published version of the manuscript.

## CONFLICT OF INTEREST

The authors declare no conflict of interest.

## Data Availability

The data that support the findings of this study are available from the corresponding author upon request.
